# Draft Genome Sequences of Five *Pseudomonas aeruginosa* Clinical Strains Isolated from Sputum Samples from Cystic Fibrosis Patients

**DOI:** 10.1128/genomeA.01528-15

**Published:** 2016-01-28

**Authors:** M. B. Couger, Anna Wright, Erika I. Lutter, Noha Youssef

**Affiliations:** Department of Microbiology and Molecular Genetics, Oklahoma State University, Stillwater, Oklahoma, USA

## Abstract

We report here the draft genome sequences of five *Pseudomonas aeruginosa* isolates obtained from sputum samples from two cystic fibrosis patients with chronic colonization. These closely related strains harbor 225 to 493 genes absent from the *P. aeruginosa* POA1 genome and contain 178 to 179 virulence factors and 29 to 31 antibiotic resistance genes.

## GENOME ANNOUNCEMENT

The genomes of five *Pseudomonas aeruginosa* isolates (strains 14649, 14650, 14651, 14672, and 14673), obtained at the Adult Cystic Fibrosis Clinic in Calgary, Alberta, Canada, from sputum samples from two cystic fibrosis patients with chronic colonization, were sequenced using the Illumina MiSeq platform with 300 × 2 paired-end chemistry and an average library insert size of 700 bp. Standard Illumina quality filtering settings were used for base calling. The sequence data from each clinical isolate were assembled using Velvet (1, 2), using a *k*-mer value of 101 bp and a minimum coverage of 7×. The gene models for each isolate were created using the prokaryotic gene calling software package Prodigal (3). All protein sequences from each isolate genome were functionally annotated using a combination of NCBI Blast C++ homology search (4) and HMMER 3.0 hmmscan (5, 6) against the Pfam 26.0 database (7).

Assembly *N*_50_ contig values of 489,659, 417,833, 308,331, 427,030, and 519,426 bp; genome sizes of 6.23, 6.37, 6.67, 6.36, and 6.36 Mbp; and G+C contents of 66.4%, 66.5%, 66.1%, 66.5%, and 66.5% were obtained for strains 14649, 14650, 14651, 14672, and 14673, respectively. A comparison of the genomes to the reference sequence of *P. aeruginosa* strain PAO1 (8) revealed strain-specific differences. The genomes of strains 14649, 14650, 14651, 14672, and 14673 contained 225, 229, 493, 230, and 227 genes, respectively, that were absent from the PAO1 genome. Conversely, 134, 94, 92, 102, and 107 genes were present in the reference genome PAO1 compared to the genomes of 14649, 14650, 14651, 14672, and 14673, respectively (see Table 1).

**TABLE 1:** General genomic features of the five *P. aeruginosa* genomes sequenced in this study

*P. aeruginosa* strain	Results of genome assemblies			No. of genes^a^:		Results from comparisons to VFDB and ARDB		
	Genome size (Mb)	*N*_50_ contig value (bp)	G+C content (%)	Present in isolate genome, absent in reference genome	Absent in isolate genome, present in reference genome	No. of virulence factor genes	No. of antibiotic resistance genes	GenBank accession no.
14649	6.23	489,659	66.4	225	134	178	31	LKPS00000000
14650	6.37	471,833	66.5	229	94	179	31	LKPT00000000
14651	6.67	308,331	66.1	493	92	179	29	LKPU00000000
14672	6.36	427,030	66.5	230	102	179	31	LKPV00000000
14673	6.36	519,426	66.5	227	107	179	26	LKPW00000000

a Results of BLASTP against PAO1 reference genome.

Protein models important to pathogenesis were identified using comparisons to the Virulence Factor Database (VFDB) (9), and antibiotic resistance genes were identified using the ARDB database (10). The genomes of strains 14649, 14650, 14651, 14672, and 14673 contained 178, 179, 179, 179, and 179 virulence factors and 31, 31, 29, 31, and 26 antibiotic resistance genes (see Table 1). These genes included those for β-lactamase, multidrug resistance efflux pumps (11), bacitracin resistance protein (12), and the efflux transporter system OprM (13).

### Nucleotide sequence accession numbers.

The GenBank accession numbers for the genomes of 14649, 14650, 14651, 14672, and 14673 are listed in Table 1.
